# Use of vitamins by participants in amyotrophic lateral sclerosis clinical trials

**DOI:** 10.1371/journal.pone.0237175

**Published:** 2020-08-13

**Authors:** Tino Prell, Julian Grosskreutz, the Pooled Resource Open-Access ALS Clinical Trials Consortium

**Affiliations:** 1 Hans Berger Department of Neurology, Jena University Hospital, Jena, Germany; 2 Center for Healthy Ageing, Jena University Hospital, Jena, Germany; University of Florida, UNITED STATES

## Abstract

Patients’ vitamin intake is often not documented and is therefore not considered sufficiently in studies of prescribed medication in patients with amyotrophic lateral sclerosis (ALS). We aimed to determine the prevalence of vitamin use by participants in ALS clinical trials. Data about demographics, disease severity (ALS Functional Rating Scale) and concomitant medication were obtained from the Pooled Resource Open-Access ALS Clinical Trials Database, which contains records from more than 6000 ALS patients who participated in 23 phase II/III clinical trials. Information about vitamin intake for all study subjects was coded into major categories. Clinical data of vitamin users and nonusers were compared, and regression analysis was used to explore the associations among clinical parameters, vitamin use and two measures of disease progression. From the 40.996 available medication records from 6274 subjects, 7338 (17.9%) concerned vitamins. One or more vitamins were used by 3331 subjects (53.1%). Most common was vitamin E, vitamin C and multivitamins. Patients who did and did not take vitamins did not differ in terms of disease progression and ALS Functional Rating Scale score. Patients who took vitamins were younger, were more often female, had a shorter time between onset and diagnosis, had shorter disease duration and more frequently had limb-onset types. Disease progression rate and disease aggressiveness were not associated with vitamin use. Despite unclear evidence, the use of vitamins in ALS is common. However, rapid progression was not observed to be associated with vitamin use.

## Introduction

Amyotrophic lateral sclerosis (ALS) is a severe neurodegenerative disorder that is characterised by progressive muscle atrophy, respiratory failure and several nonmotor symptoms [1]. Despite many clinical trials, to date there is no cure for ALS and mean length of survival after onset ranges from 2 to 5 years [2]. Naturally, people with ALS have an interest in alternative therapies and drugs, even though there is no evidence of the efficacy of these therapies. However, if these alternative therapies do have an effect on disease progression, they may influence the validity of clinical trials. Prescribed or experimental drugs may also interact. In addition, an imbalance in study conditions could occur: for example, if alternative therapies are preferred by a certain subgroup of patients. Thus for many reasons, the alternative therapies and over‐the‐counter (OTC) medications used by people with ALS, and how often they are used, should be investigated. One methodological problem, however, is that OTC medications are often not regularly recorded by doctors and are therefore not considered sufficiently in investigations of prescribed medication [3]. The Pooled Resource Open-Access ALS Clinical Trials (PRO-ACT) database is the ideal tool for such investigations. The PRO-ACT database is the largest publicly available repository of merged data from ALS-related clinical trials [4]. According to good clinical practice at the beginning and during a study, all drugs (prescribed and OTC) must be recorded. We explored the prevalence of vitamin use by participants in ALS clinical trials.

## Materials and methods

Data used in this study were obtained from the PRO-ACT database (https://nctu.partners.org/ProACT/). This database contains records from more than 6000 patients with ALS who participated in 23 phase II/III clinical trials. Different types of information may be available for different patients because the data from multiple trials were merged. Among other demographic data, family history, laboratory data, respiratory function, disease severity and concomitant medication are available in PRO-ACT. Concomitant medication includes the medications given to the patients during a trial, such as drugs for patients’ other conditions, supplements favoured by the patients and medication related to any adverse events caused by the treatment. Neither consent nor ethical approval was sought for our analysis because this data set was publicly available.

From the dataset we derived scores on the ALS Functional Rating Scale (original 10 items, 40 points: 40-ALSFRS), scores on the revised ALSFRS (12 items, 48 points: 48-ALSFRS-R), disease duration (months from disease onset to study entry), time (months) between onset and diagnosis, age, sex, site of onset (bulbar or limb) and medication. Disease progression was calculated as the 40-ALFRS score divided by disease duration (months) or as the 48-ALSFRS-R score divided by disease duration (months) until the first available ALSFRS-R score. We used the D50 [5,6], a summative descriptor of disease aggressiveness, to derive the estimated time until the ALSFRS or ALSFRS-R score declined to 20 or 24 points, respectively. Only the medications taken before enrollment into the distinct studies were analysed; changes of medication during the ongoing study were not included. Therefore, we used 40.996 medication records from 6274 patients with ALS to determine the prevalence of vitamin use. The association with clinical variables were analysed in 4746 subjects for whom clinical data were available.

The information about vitamins for all study subjects was coded by one of the authors (TP) into major categories: multivitamins and vitamins A, B1, B6, B12, C and so forth. The categories were not mutually exclusive; some subjects took different vitamins simultaneously. Doses and frequencies were not consistently reported in PRO-ACT and could therefore not be analysed.

The SPSS statistical software package (version 25.0; IBM Corporation, Armonk, NY, USA) was used for all statistical analyses. Normality was tested with the Shapiro-Wilk test. The chi‐square test, *t* test or Mann–Whitney *U* test and 95% confidence intervals were used to compare the demographic and clinical characteristics of patients who took vitamins with those of patients who did not. A linear regression analysis was used to explore the association between clinical parameters (age, gender, length of time between onset and diagnosis, use of riluzole [yes/no], onset type [bulbar/limb] and vitamin use [yes/no]) and either disease progression rate or D50 (Akaike’s information criterion with stepwise forward selection). Statistical significance was set at *P* < 0.01.

## Results

Details of the PRO‐ACT cohort have been reported previously [4]. Records of medication taken before study entry were available from 6274 patients with ALS. Clinical parameters, which were unavailable for some subjects, are listed in Table 1.

**Table 1:** Characteristics of participants.

**Characteristic**		**N**	**%**	**Number of subjects with available data**
Gender	Female	1789	37.7	4746
	Male	2957	62.3	
Onset type	Bulbar	2588	54.5	4746
	Limb	2158	45.5	
**Characteristic**		**Median**	**IQR**	**Number of subjects with available data**
ALSFRS score		31.0	7.0	3848
ALSFRS-R score		39.0	7.0	1714
Age (years)		56.0	17.0	4746
Progression rate		0.46	0.50	4746
D50		31.4	28.8	4746
Time from onset to diagnosis (months)		9.5	9.5	4746
Disease duration (months)		18.0	17.5	4746

ALSFRS: Amyotrophic Lateral Sclerosis Functional Rating Scale; ALSFRS-R: ALSFRS revised; IQR: interquartile range. No metric values were normally distributed.

**Fig 1:**
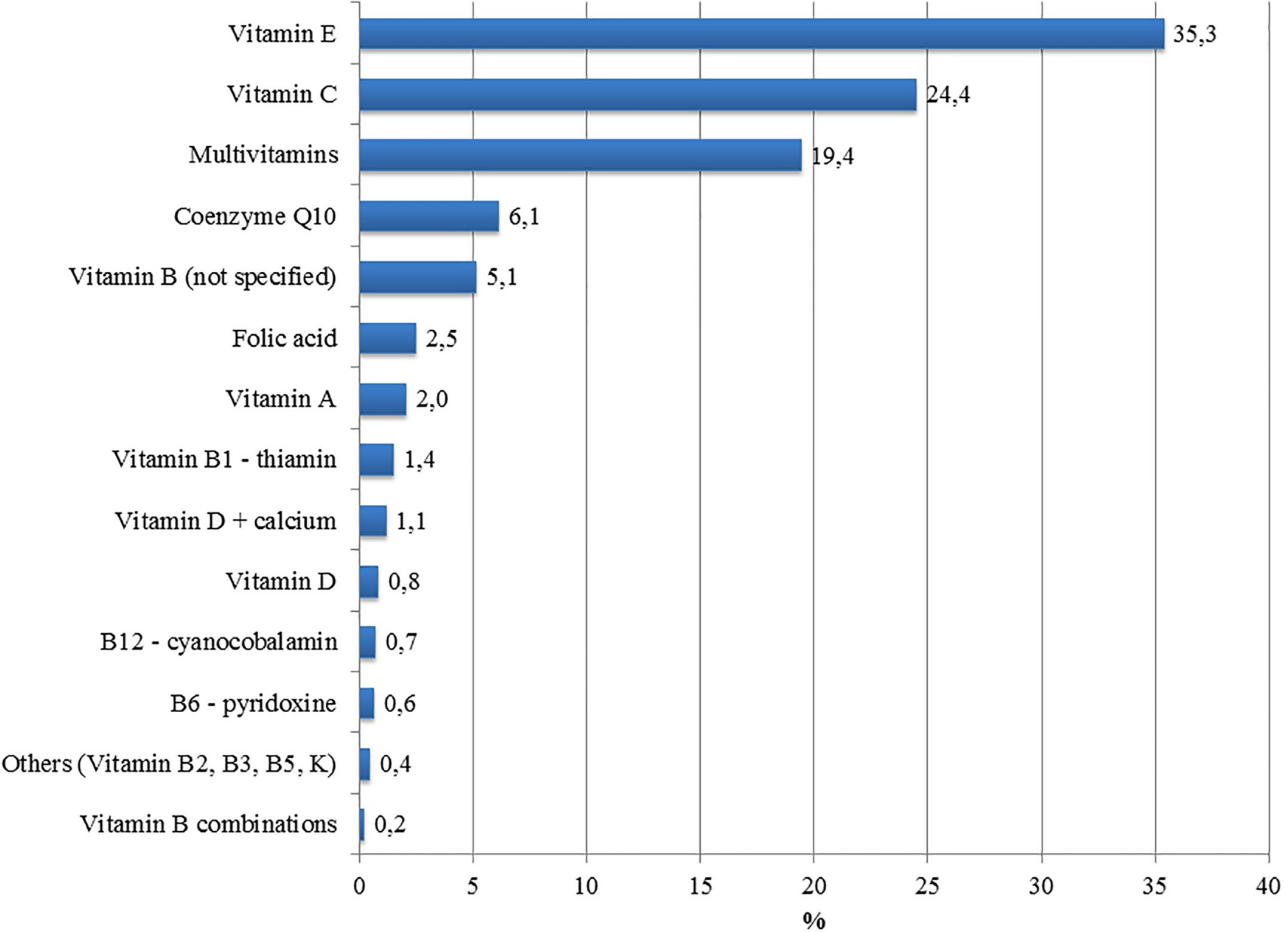
Frequency (%) of vitamin use in the medication records, including any vitamin (N = 7338).

Vitamin use was common. Of the 40.996 available medication records, 7338 (17.9%) included information about vitamins. The most common vitamins taken were vitamin E, vitamin C and multivitamins (Fig 1). One or more vitamins were used by 3331 subjects (53.1% of 6274 patients).

With regard to the 95% confidence intervals and results from the Mann–Whitney *U* test, the patients who did and did not take vitamins did not differ in terms of disease progression rate and ALSFRS-R scores. However, patients who took vitamins were younger, had a shorter time between onset and diagnosis, and had a shorter disease duration after the first symptom; female patients who took vitamins outnumbered their male counterparts; and limb-onset types were more common among patients who took vitamins (Table 2).

**Table 2:** Comparison between vitamin users and vitamin nonusers.

**Characteristic**	**No vitamin use**		**Vitamin use**		
	**Median**	**95% CI**	**Median**	**95% CI**	***P***
ALSFRS score	30.00	30.00–31.00	31.00	31.00–32.00	<0.001
ALSFRS-R score	39.00	39.00–40.00	39.00	39.00–40.00	0.78
Age (years)	57.00	57.00–58.00	56.00	56.00–57.00	0.007
Disease progression rate	0.48	0.46–0.50	0.47	0.45–0.48	0.31
D50	31.94	31.00–33.03	32.22	31.27–33.06	0.65
Time from onset to diagnosis (months)–	13.64	12.69–14.46	11.77	11.28–12.00	<0.001
Disease duration (months)	19.48	18.85–20.30	18.16	17.67–18.62	0.001
**Characteristic**	**N**	**%**	**N**	**%**	***P***
Gender					<0.001
Female	653	38.5	1136	37.2	
Male	1041	61.5	1916	62.8	
Onset type					<0.001
Bulbar	1052	62.1	1536	50.3	
Limb	642	37.9	1516	49.7	

CI, confidence interval; ALSFRS: Amyotrophic Lateral Sclerosis Functional Rating Scale; ALSFRS-R: ALSFRS revised.

Disease progression rate was associated with time between onset and diagnosis, gender and age but not with vitamin use, riluzole use, or onset-type (corrected R^2^ = 0.16, *P* < 0.001; Table 3). Also, the D50 was associated with time between onset and diagnosis, gender and age but not with vitamin use, riluzole use, or onset-type (corrected R^2^ = 0.14, *P* < 0.001; Table 3).

**Table 3:** Linear regression analysis: Predictors of disease progression.

Dependent variable	Unstandardised coefficients		Standardised coefficients	*P*
	b	Standard error	β	
Disease progression rate*				
Constant	0.773	0.035		<0.001
Time from onset to diagnosis	−0.018	0.001	0.965	<0.001
Gender (female)	0.074	0.015	0.026	<0.001
Age	0.002	0.001	0.008	0.006
D50*				
Constant	32.242	2.672		<0.001
Time from onset to diagnosis	1.247	0.045	0.938	<0.001
Gender (male)	−7.102	1.125	0.050	<0.001
Age	−0.147	0.047	0.012	0.002

*Higher progression rate and lower D50 indicate rapid progression.

## Discussion

The main vitamins taken by participants with ALS were vitamin E, vitamin C and multivitamins. Vitamins were taken more commonly by younger participants and female participants. Vitamin use was not independently associated with disease progression rate or disease aggressiveness. This indicates that rapid progression is not a general reason to use vitamins in ALS.

It is not surprising that people suffering from a deadly neurodegenerative disorder are willing to use complementary therapy. However, what is the reason for taking vitamins without clear evidence of their efficacy? Vitamin E was the vitamin most commonly used by the PRO-ACT cohort. Vitamin E is an important cellular antioxidant. The analysis of five prospective cohort studies of more than one million persons suggested that long-term use of vitamin E could be inversely associated with risk of ALS [7]. Although promising in an ALS mouse model [8], vitamin E supplementation was found to be ineffective in randomised trials of humans with ALS [9,10]. Nevertheless, there have been and still are recommendations, especially on the Internet, for people with ALS to take vitamin E [11]. Despite low‐quality evidence, patients with ALS may also consider using vitamin E for the treatment of cramps [12]. The reasons why patients took vitamin E are not provided by the PRO-ACT database.

The second and third vitamins most commonly used were vitamin C and multivitamins. A pooled analysis of the results of five large prospective studies showed that neither supplemental use nor high dietary intake of vitamin C was associated with ALS risk [13]. A recent retrospective study of 1585 patients with random decision forests demonstrated that use of vitamin A and multivitamins were predictive of survival duration, which emphasises that for patients with ALS, clinicians should focus on prophylaxis against health complications with nutritional supplements, vitamins and alternative herbs [14].

Vitamins are generally well tolerated and, with few exceptions, do not cause serious adverse effects [15]. This probably explains their wide use by people with ALS, who face a progressive, fatal condition. Most vitamins are available without prescription; therefore, daily use of these OTC medications are not recorded routinely and are not considered in investigations of medication prescribed for ALS. Some vitamins (thiamin, vitamin B12, vitamin K) have minor and reversible adverse effects; others, such as vitamins A, E, D, can cause serious adverse events [16].

This study had some limitations. Because the PRO-ACT database contains data only from clinical trials with very specific entry criteria for patients, a selection bias in favour of prevalent cohorts might exist. This limits the generalisability of the results. Moreover, in contrast to the general population of patients with ALS, those who participate in clinical trials tend to be younger, limb onset is more common, and the prognosis is better [4]. Although the PRO-ACT database reflects a large sample, full clinical data (e.g., ALSFRS) were not available for the entire cohort of 6274 subjects. Therefore, further investigations in other real world datasets are necessary to document the high prevalence of vitamin use among patients with ALS.
